# Isolated hair loss on the eyebrow: five cases with trichoscopic features^☆^

**DOI:** 10.1016/j.abd.2021.04.015

**Published:** 2022-03-07

**Authors:** Güldehan Atış, Ayşenur Şam Sarı, Pembegül Güneş, Cansu Sönmez

**Affiliations:** a Department of Dermatology, Haydarpaşa Numune Training and Research Hospital, Hamidiye Medicine Faculty, University of Health Sciences, İstanbul, Turkey; b Department of Pathology, Memorial Hospital, İstanbul, Turkey; c Department of Pathology, Haydarpaşa Numune Training and Research Hospital, Hamidiye Medicine Faculty, University of Health Sciences, İstanbul, Turkey

**Keywords:** Alopecia, Dermoscopy, Eyebrows, Trichotillomania

## Abstract

Alopecia areta (AA) and trichotillomania (TTM) are common causes for hair loss on the eyebrows. Yellow dots, vellus hairs, anisotrichosis, empty follicular openings, and black dots were observed in the present study’s patients with AA. Split hairs, question mark hairs, broken hairs, flame hairs, black dots, hairs with different lengths, and hemorrhagic areas were found in the patients with TTM. Trichoscopy is a very useful and helpful technic in distinguishing AA and TTM on the eyebrows.

## Introduction

Eyebrow loss might be due to trauma, infections, autoimmune, neoplastic, genetic conditions, and various dermatoses.^1^ The authors presented five cases with eyebrow loss to emphasize the distinguishing features of two clinically confusing entities.

## Case Report

**Figure 1:**
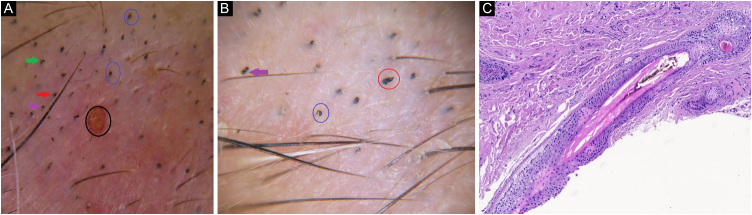
(A), Hemorhagic area (black circle), split hair (red arrow), question mark hairs (gren arrows), black dots (blue circle). (B), Broken hair (purple arrow), flame hair (red circle), black dots (blue circle). (C), Inner rooth sheath distruption, and intrafollicular pigment cast (Hematoxylin & eosin, ×100).

Case 1 A 60-year-old woman was presented with marked eyebrow loss, which had not improved with topical corticosteroids. Hairs with different lengths, hemorrhagic areas, split hairs, question mark hairs, broken hairs, flame hairs, and black dots were observed on trcihoscopy (Fig. 1A e B). Trichomalacia, ectopic, pigmented cortical cells, pigment casts in inner root sheath and in follicles were seen on histopathology (Fig. 1C). She was diagnosed with Trichotillomania (TTM), consulted with a psychiatrist, and was diagnosed as a generalized anxiety disorder. Fluoxetin and risperidone were prescribed.

**Figure 2:**
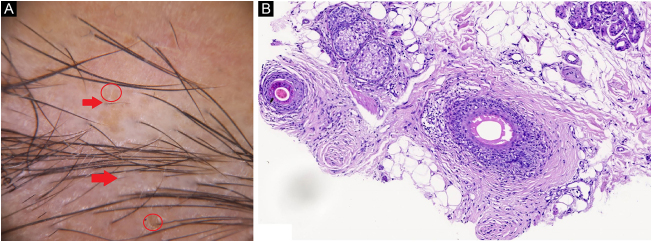
(A), Vellus hairs (red arrow), yellow dots (red circle). (B), Lymphocytic infiltrate with peribulbar fibrosis (Hematoxylin & eosin, ×100).

Case 2 A 32-year-old woman presented with her third episode of eyebrow loss during one year period. Vellus hairs, yellow dots, and anisotrichosis were detected on trichoscopy (Fig. 2A). There was lymphocyte invasion of the outer root sheath, and a lymphocytic infiltrate with peribulbar fibrosis on histopathology (Fig. 2B). The patient was diagnosed with Alopecia Areata (AA). 5 mg/ml triamcinolone acetonide was injected intralesionally per month three times. At the end of the third month, the hairs regrew.

**Figure 3:**
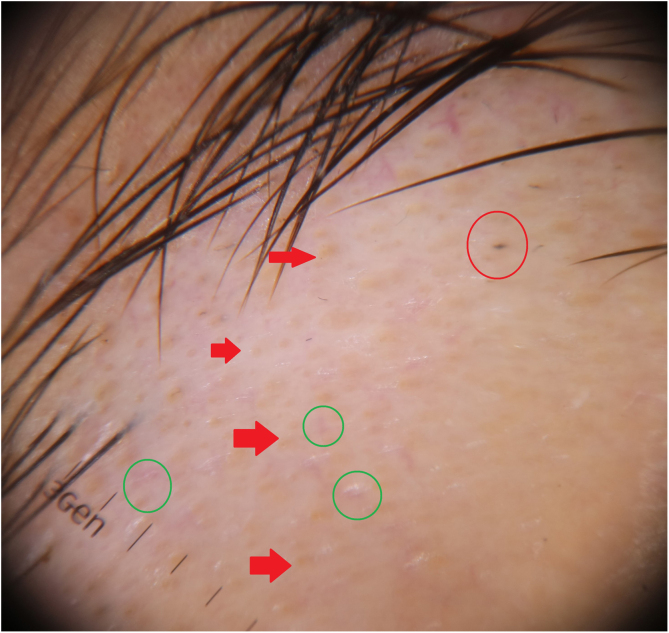
Yellow dots (red arrow), empty follicular openings (gren circle), and black dot (red circle).

Case 3 An 18-year-old man applied to the outpatient clinic with eyebrow loss for five months. He had had hair loss on the scalp six months ago that spontaneously regrew up. There were alopecic areas on the right eyebrow; and numerous yellow dots, empty follicular openings, and a few black dots on trichoscopy (Fig. 3). The patient was diagnosed with AA. Intralesional triamcinolone acetonide (5 mg/ml) was injected once, and the follow-up continues.

**Figure 4:**
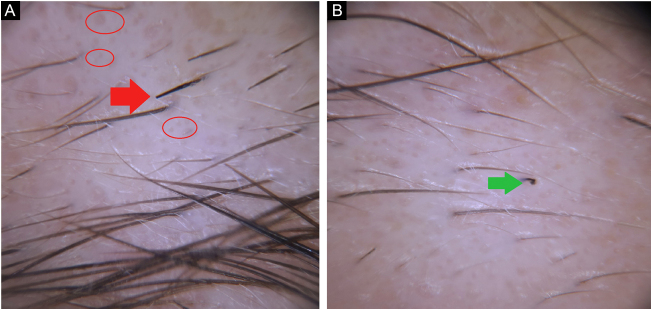
(A), Empty follicles (red circle), broken hair (red arrow). (B), Question mark hair (green arrow).

Case 4 A 31-year-old woman was presented with a five-month-history of eyebrow loss. Spontaneous regrowth of eyebrow hairs happened after a complete loss. She denied any emotional stress or conflict on her job and persistently refused to consult the psychiatrist. On trichoscopy; hairs of different lengths, empty follicles, broken hairs, question mark hairs (Fig. 4A–B) were observed. The patient was diagnosed with TTM then quit the follow-up.

**Figure 5:**
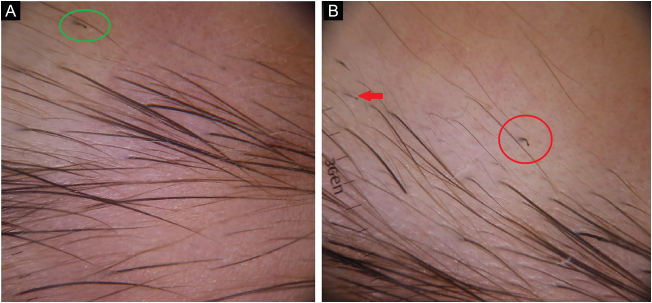
(A), Broken hair (red arrow), comma hair (red circle). (B), Tulip hair (green circle).

Case 5 A 10-year-old girl presented with eyebrow loss for two months, which begun after she had had a serious emotional stress. Her mother mentioned seeing her daughter pulling her eyebrows. Her eyebrow hairs were sparse; and broken hairs, comma hairs, tulip hairs, and black dots were observed on trichoscopy (Fig. 5A–B). The patient was diagnosed with TTM and referred to a child and adolescent psychiatrist.

## Discussion

Trichotillomania is characterized by pulling one’s own hair repetitively in a compulsive manner.^2^ It is classified inside the obsessive-compulsive disorder and related disorders in the Diagnostic and Statistical Manual of Mental Disorder-Fifth Edition (DSM-5).^3^ Before pulling their hair out, patients experience anxiety; and feel relaxed thereafter.^4^ Patients with TTM are usually misdiagnosed as AA due to their episodic nature.^4^ Therefore, they are treated unsuccessfully as the study’s first case.

Alopecia areata is characterized by T-cell infiltration of the hair bulb. It rarely represents isolated eyebrow involvement^1^ and may rarely appear as an initial finding of AA.^5^

Although trichoscopic features of TTM on the scalp were well described, trichoscopy of eyebrow TTM were discussed in limited reports.^2^ On trichoscopy of eyebrows, the authors observed hairs with different lengths, split hair, question mark hair, broken hair, flame hair, comma hair, tulip hairs, black dots, hyperpigmented and hemorrhagic areas on the surface. Yellow dots are one of the common features in AA and show a regular distribution,^6^ but rare in TTM, and the distribution is irregular.^7^ In the AA patients, the authors observed a few irregularly distributed yellow dots, but the authors didn't detect them in TTM patients. It may be due to insufficient space for trichoscopic examination in alopecic areas of the eyebrows which may justify the absence in the presented case with TTM. Empty follicles are also one of the trichoscopic features of AA and are more common on eyebrows. This might be associated with the brevity of the eyebrow hair cycle.^5^ The authors think that washing the face more frequently than the scalp may contribute to this situation. Sebum, keratin, and broken hairs inside the follicle openings might be removed by washing. In one of the study’s AA patients, the authors observed numerous empty follicles. Also, the evaluation of multiple trichoscopic images together contributes to define the diagnosis. Khunkhet et al. observed trichoscopic features of TTM and AA on the scalp and they evaluated five images for each patients.^8^

In a study, exclamation mark hairs and tapered hairs are claimed as common features of the eyebrow AA. However, they are observed less common than scalp AA.^6^ Despite that, the authors did not observe exclamation marks or tapered hairs in the AA patients. It may be due to a low number of cases. Vellus hairs are asserted as a common feature of eyebrow AA.^5^ The authors detected vellus hairs in one of the AA patients.

Broken hairs, are transverse fractures of the terminal hair shaft due to inflammation or rapid regrowth of black dots. Broken hairs and black dots are also common features of both scalp and eyebrow AA.^5^ The authors observed those in both of the AA patients.

In conclusion, although trichoscopic features of AA and TTM of the eyebrows are similar to those of the scalp, there are some minor differences. It should be remembered that trichoscopy is very useful in distinguishing AA and TTM on the eyebrows.

## Financial support

None declared.

## Authors' contributions

Güldehan Atış: Approval of the final version of the manuscript; critical literature review; data collection, analysis, and interpretation; effective participation in research orientation; ıntellectual participation in propaedeutic and/or therapeutic management of studied cases; critical manuscript review; preparation and writing of the manuscript; study conception and planning.

Ayşenur Şam Sarı: Approval of the final version of the manuscript; critical literature review; data collection, analysis, and interpretation; effective participation in research orientation; ıntellectual participation in propaedeutic and/or therapeutic management of studied cases; critical manuscript review; preparation and writing of the manuscript.

Pembegül Güneş: Approval of the final version of the manuscript; data collection, analysis, and interpretation; effective participation in research orientation; critical manuscript review.

Cansu Sönmez: Approval of the final version of the manuscript; effective participation in research orientation; critical manuscript review.

## Conflicts of interest

None declared.
